# Time-Restricted Feeding Improves Glucose Tolerance in Rats, but Only When in Line With the Circadian Timing System

**DOI:** 10.3389/fendo.2019.00554

**Published:** 2019-08-21

**Authors:** Paul de Goede, Ewout Foppen, Wayne I. G. R. Ritsema, Nikita L. Korpel, Chun-Xia Yi, Andries Kalsbeek

**Affiliations:** ^1^Laboratory of Endocrinology, Amsterdam University Medical Center, Amsterdam Gastroenterology & Metabolism, University of Amsterdam, Amsterdam, Netherlands; ^2^Hypothalamic Integration Mechanisms Group, Netherlands Institute for Neuroscience (NIN), An Institute of the Royal Netherlands Academy of Arts and Sciences, Amsterdam, Netherlands; ^3^Department of Endocrinology and Metabolism, Amsterdam University Medical Center, University of Amsterdam, Amsterdam, Netherlands

**Keywords:** feeding behavior, metabolism, intravenous glucose tolerance test (ivGTT), insulin sensitivity, shift-work, Type 2 diabetes mellitus (T2DM)

## Abstract

Epidemiological studies indicate that shift-workers have an increased risk of type 2 diabetes mellitus (T2DM). Glucose tolerance and insulin sensitivity both are dependent on the circadian timing system (i.e., the time-of-day) and fasting duration, in rodents as well as humans. Therefore, question is whether manipulation of the circadian timing system, for example by changing the timing of feeding and fasting, is a potential preventive treatment for T2DM. Time-restricted feeding (TRF) is well-known to have profound effects on various metabolic measures, including glucose metabolism. However, experiments that directly measure the effects of TRF on glucose tolerance and/or insulin sensitivity at different time points throughout the 24 h cycle are lacking. Here we show, in rats, that TRF in line with the circadian timing system (i.e., feeding during the active phase) improves glucose tolerance during intravenous glucose tolerance tests (ivGTT) in the active phase, as lower insulin levels were observed with similar levels of glucose clearance. However, this was not the case during the inactive phase in which more insulin was released but only a slightly faster glucose clearance was observed. Contrasting, TRF out of sync with the circadian timing system (i.e., feeding during the inactive phase) worsened glucose tolerance, although only marginally, likely because of adaptation to the 4 week TRF regimen. Our results show that TRF can improve glucose metabolism, but strict adherence to the time-restricted feeding period is necessary, as outside the regular eating hours glucose tolerance is worsened.

## Introduction

An increasing number of people are suffering from type 2 diabetes mellitus (T2DM) (1). T2DM is characterized by hyperglycemia resulting from insulin resistance. The main risk factors for T2DM are excessive caloric intake and a lack of exercise; however, other factors such as disturbed sleep/wake rhythms may also contribute to disease development [reviewed in (2, 3)]. Disturbed sleep/wake rhythms are especially pronounced in people performing shift work, as they often are awake during the natural resting phase, sleep during daytime and eat at irregular times. With modern societies increasingly relying on shift-work a better understanding of the effects of shift-work on glucose metabolism, but also health in general, is essential. A widely used animal model to study the metabolic effects of shift-work is time-restricted feeding (TRF) in which the opportunity to eat, but not the amount, is restricted to a certain period of the day [reviewed in (4, 5)]. In nocturnal animals such as mice and rats the chosen period is usually (part of) the light period (=inactive phase) to mimic shift work and (part of) the dark period (=active phase) as a control condition. TRF to the active phase is associated with health benefits, whilst TRF to the inactive phase is associated with negative health effects (6). It has long been known that glucose tolerance displays clear day/night differences. Nevertheless, glucose tolerance tests (GTT) to study the effects of TRF on glucose metabolism are usually only performed at one time point (7–10). Therefore, it is not clear how much of the variation found is due to (changes in) the circadian timing system (11) or to differences in the preceding fasting period. In order to separate the effects of diurnal variation due to the intrinsic timing system from that of TRF we designed our experiment in such a way that all animals were tested both 4 h after the onset of the dark phase and 4 h after the onset of the light phase. By choosing two time points 12 h apart also fasting duration before each GTT was counter-balanced between the light-TRF and dark-TRF groups. As TRF during the active phase is associated with metabolic health improvements and TRF during the inactive phase with negative health effects we hypothesized that animals fed only during the dark/active period would show improved glucose tolerance, whilst animals only fed during the light/inactive period would show impaired glucose tolerance.

## Materials and Methods

### Animals and Housing

**Figure 1:**
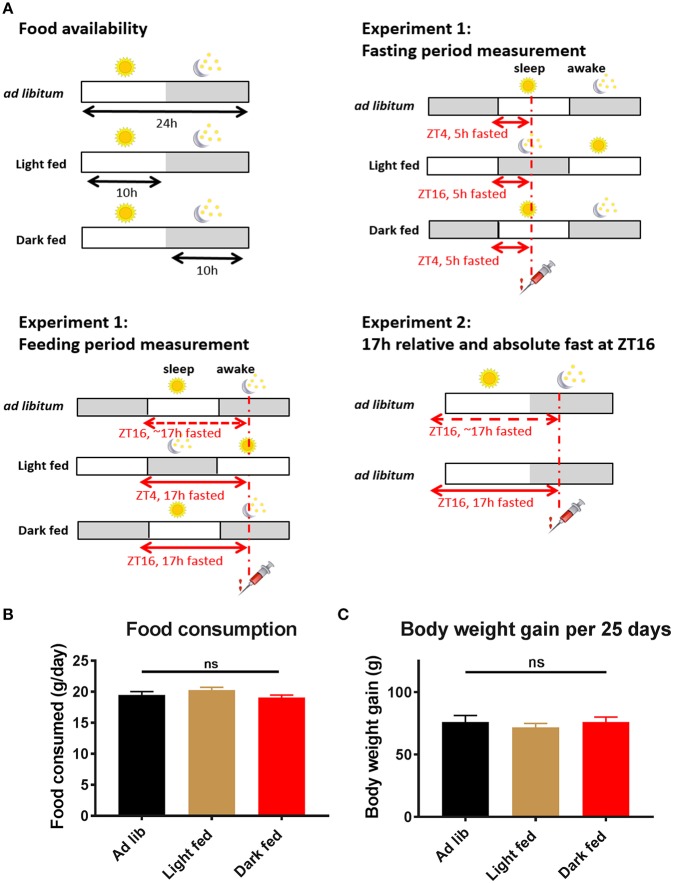
Experimental design and basic physiological measures of the rats. **(A)** Experimental design of Experiment-1 and Experiment-2. Time-restricted fed animals had daily access to chow pellets for 10 h during either the light phase (ZT1-11) or the dark phase (ZT13-23). Three and four weeks after the start of the TRF protocol an intravenous glucose tolerance test (ivGTT) was performed. On these experimental days an ivGTT was performed at ZT4 or ZT16, during which blood samples were taken just before a glucose bolus injection at *t* = 0 as well as at *t* = 5, 10, 20, 30, and 60 min. All animals were fasted for at least 5 h on the experimental days, but as TRF animals remained on their assigned Feeding regimen during the experimental days they were effectively fasted for 17 h during the Feeding period measurement (i.e., dark fed animals were 17 h fasted during the measurement at ZT16, whilst light fed animals were fasted for 17 h during the measurement at ZT4). **(B)** Daily food consumption in the test weeks did not significantly differ between the 3 experimental groups (average of 2 days in week 3 and 2 days in week 4, *p* = 0.23; one-way ANOVA, *n* = 14–16 per group). **(C)** Body weight gain in the period between the start of the TRF regimen and the 4th week of the TRF protocol did not significantly differ between the 3 groups (*p* = 0.73; one-way ANOVA, *n* = 14–16 per group).

Forty-five male Wistar rats with a starting weight of ≈280 g (Charles River) were used for the TRF intravenous glucose tolerance test (ivGTT) experiments. An additional eight animals fed *ad libitum* were tested with two different fasting durations. Animals were housed at a constant temperature of 22°C under a controlled 12:12 light:dark cycle, lights on at Zeitgeber Time 0 (ZT0) and lights off at ZT12. After arrival to the institute animals had an acclimation period of 1 week after which they were individually housed and randomly assigned to one of three TRF groups for 4 weeks: *ad libitum* feeding (AL), light phase feeding (light-TRF), or dark phase feeding (dark-TRF) (*n* = 14–16 per group). Light-TRF and dark-TRF animals had access to chow pellets for 10 h in the middle of the light or dark phase, respectively (Figure 1A). In automated cages food access was controlled by a vertically moving metal plate that completely blocked access to the food bin, alike an old castle gate. All animals had *ad libitum* access to tap water. After 2 weeks of TRF, a jugular vein surgery was performed as described previously (11). Animals could recover from the surgery for 1 week, whilst remaining on their assigned feeding conditions. All experiments were approved by the Dutch government and performed in accordance with the guidelines on animal experimentation of the Netherlands Institute for Neuroscience.

### Experimental Procedure

#### TRF ivGTT Experiment (Experiment-1)

After 3 and 4 weeks on TRF an ivGTT was performed at either ZT4 or ZT16, i.e., 4 h after lights-on or 4 h after lights-off, in a randomized order. During the experimental days all animals remained on their assigned TRF conditions, but in addition for all animals food was removed 5 h before the ivGTT (if applicable). Consequently, dark fed animals were fasted for 17 h during the ZT16 measurement and light fed animals were fasted for 17 h during the ZT4 measurement (Figure 1A). During the other experiment, i.e., ZT4 in dark-TRF and ZT16 in light-TRF, animals were fasted for 5 h. Fasting periods of the *ad libitum* fed animals were comparable to those of the dark fed animals, although the 17 h fasting period for the ZT16 time point is not absolute, as animals might have eaten a little during the second half of the light period before food was removed at ZT11. After a baseline blood sample (*t* = 0 min) had been taken animals were infused with glucose (1 mg/g bodyweight, dissolved in saline) and blood samples were taken again 5, 10, 20, 30, and 60 min after the glucose infusion. Typically, 0.25 ml of blood was drawn during a sample.

#### Ad Libitum Control Experiment (Experiment-2)

After recovery from surgery the extra eight *ad libitum* fed rats were randomly assigned to either a 5 h or a 17 h fast before the start of an ivGTT in the dark, i.e., awake, period (ZT16). After a recovery period of 1 week the rats were tested again with the other fasting duration. The ivGTT procedure itself was identical to the one described above for the TRF ivGTT experiments.

### Glucose and Insulin Measurements

Blood glucose was measured directly at each sampling point during the ivGTT from the untreated blood samples using blood glucose test strips with a 0.1 mmol/L accuracy (FreeStyle, Abbott Diabetes Care). Plasma insulin was measured using a radioimmunoassay (Millipore).

### Statistics

All data are represented as means ± SEM. Two-way repeated measure (RM) ANOVAs were used to test for the effects of *TRF* and *Sampling* (i.e., *t* = 0, *t* = 5, *t* = 10, etc. after glucose infusion) as well as the *Interaction* (*TRF* ^*^ *Sampling*) for the insulin and glucose profiles during the ivGTTs. Tukey's *post-hoc* tests were performed to compare the three different TRF groups. Delta values for glucose and insulin concentrations were determined by subtracting the baseline value (*t* = 0) from the value of each of the subsequent time points. The net AUC (i.e., negative AUC [“undershoot”] subtracted from positive AUC) was determined using these delta values and the trapezoid rule for the duration of the entire ivGTT measurement (0–60 min). Two-way ANOVAs were used to test for effects of *TRF* (*ad libitum*, light fed and dark fed), *Fasting/Feeding* (Fasting period or Feeding period) and *Interaction* (TRF ^*^ Fasting/Feeding) on the glucose and insulin responses (expressed as net AUC). *T*-tests were performed to compare the net AUC between two different time points, i.e., ZT4 and ZT16, within each TRF group. One-way ANOVAs were used to test for differences in body weight and food intake in the experimental weeks as well as to compare the net AUC of the 17 h fasted animals at ZT16 (**Figure 3**). All statistics were run by GraphPad Prism 7.

## Results

### TRF Experiment (Experiment-1)

#### Body Weight and Food Intake

In the experimental weeks, the animals from the 3 different TRF groups ate ~19.5 g of chow/day and no significant differences in food intake were found between the groups (Figure 1B). Also body weight gain in the period between the start of the TRF regimen and the fourth week of the TRF protocol did not differ between the 3 groups (Figure 1C; *p* = 0.73; one-way ANOVA).

#### Glucose Responses

**Figure 2:**
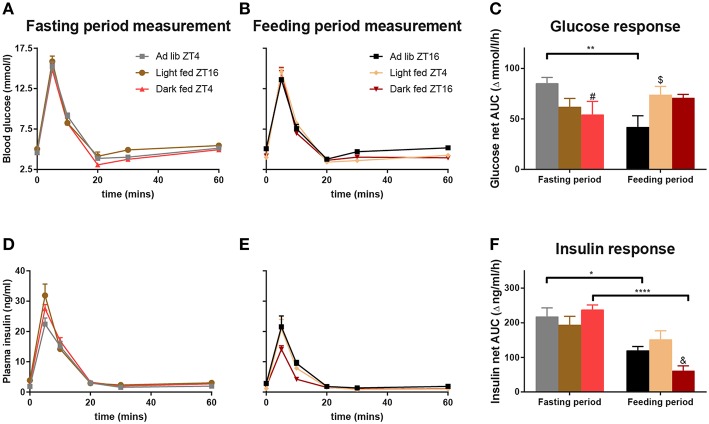
Glucose and insulin values during the ivGTTs at ZT4 and ZT16. By experimental design at any given ZT point light-TRF and dark-TRF animals always differ in their fasting status. Therefore, we chose to display the results of the ivGTT's not by ZT but according to the Feeding status of the animals. Thus, ivGTT's performed during the Feeding period are labeled “Feeding period measurements,” i.e., ZT16 for *ad libitum* and dark fed animals and ZT4 for the light fed animals. ivGTT's performed during the fasting period are labeled “Fasting period measurements,” i.e., ZT4 for the *ad libitum* and dark fed animals and ZT16 for the light fed animals. **(A,B,D,E)** Blood glucose and plasma insulin values during the ivGTT in the Fasting period **(A,D)** and during the Feeding period **(B,E)**. **(C,F)** Net AUC (i.e., negative AUC [“undershoot”] subtracted from the positive AUC) of glucose and insulin responses, respectively. AUC values of glucose and insulin are displayed relative to their respective baseline value. Table 1 summarizes the main statistical findings for all glucose and insulin measures during the GTTs. *N* = 8–13 animals per experimental group per measurement. ^*^p < 0.05, ^**^p < 0.01, ^****^p < 0.0001, #, significant difference between the Ad lib and Dark fed group; $, significant difference between the Ad lib and Light fed groups; &, significant difference between the Dark and Light fed group.

For all three groups in the TRF experiment, the intravenously administered glucose was cleared from the circulation within 20 min at both ZT4 and ZT16, i.e., blood glucose levels had returned to baseline or were even slightly lower than baseline (Figures 2A,B). During the Fasting period no differences were found between the 3 groups nor an *Interaction* effect between the sampling points and the TRF condition (Figure 2A, Table 1). Contrasting, during the Feeding period a significant *Interaction* between *TRF* and *Sampling* was found, mostly due to a higher glucose peak at *t* = 5 min for light-TRF as compared to *ad libitum* animals. Furthermore, the net AUC for the blood glucose levels showed a significant interaction between the *Fasting/Feeding-Period* and *TRF*, but no main effects of *TRF* or the *Fasting/Feeding-Period* (Figure 2C). *Post-hoc* analyses revealed that only the AL-animals showed a significant diurnal difference in AUC. Additionally, during the Fasting period dark-TRF animals showed a lower glucose response as AL-animals and during the Feeding period light-TRF animals showed a higher glucose net AUC compared to AL-animals (*p* = 0.0383 and *p* = 0.0458, respectively).

**Table 1:** Summary of the two-way ANOVA results of the insulin and glucose measures during the ivGTTs.

**Blood/insulin profile measures**	**Sampling-points (*t* = 0, *t* = 5, etc.)**	**TRF**	**Interaction**	***Post hoc* differences (*p*-value)**
Glucose during fasting period (Figure 2A)	<0.0001	0.0836	0.3555	
Glucose during feeding period (Figure 2B)	<0.0001	0.1865	<0.0001	*t* = 0 AL > l-TRF (0.0162) *t* = 5 AL < l-TRF (0.0245) *t* = 10 d-TRF < l-TRF (0.0027) *t* = 30 AL > l-TRF (0.0134) *t* = 60 AL > l-TRF & d-TRF (0.0102 & 0.0450)
Insulin during fasting period (Figure 2D)	<0.0001	0.0364	0.0342	*t* = 5 AL < l-TRF & d-TRF (<0.0001 & 0.0320) TRF: l-TRF > AL (0.0002)
Insulin during feeding period (Figure 2E)	<0.0001	0.0089	0.0709	*t* = 5 d-TRF < AL & l-TRF (0.0006 & 0.0006) *t* = 10 d-TRF < AL & l-TRF (0.0223 & 0.0397) TRF: AL > d-TRF (0.0003)
**Net AUC measures**	**(Fasting/Feeding) period**	**TRF**	**Interaction**	
Glucose (Figure 2C)	0.5019	0.8084	0.0031	Fasting period: AL > d-TRF (*p* = 0.0383) Feeding period: l-TRF > AL (*p* = 0.0458) AL ZT4 > AL ZT16 (*p* = 0.0015)
Insulin (Figure 2F)	<0.0001	0.4962	0.0082	Feeding period: d-TRF < l-TRF (*p* = 0.0089) AL ZT4 > AL ZT16 (*p* = 0.0221) d-TRF ZT4 > d-TRF ZT16 (*p* <0.0001)

*P-values for each of the main effects as well as the interaction are given. Post hoc differences between the groups and sleep/wake measurements are also given with their corresponding p-values*.

#### Insulin Responses

For all three groups and at both ZTs a sharp rise in blood insulin concentrations was found 5 min after glucose administration (Figures 2D,E). The insulin peak during the Fasting period measurement was significantly higher for the light fed and dark fed animals when compared to the AL-animals. Additionally, the light-TRF group had overall higher insulin levels when compared to the *ad libitum* fed group (Table 1). The insulin peak during the feeding period measurement was lowest for dark-TRF animals, but did not differ between AL- and light-TRF animals. In agreement with this, the two-way ANOVA showed a main effect of *TRF* with dark fed animals having lower plasma insulin levels compared to AL animals (Figure 2E). AUC of plasma insulin values showed a significant effect of the *Fasting/Feeding-Period* as well as the *Fasting/Feeding-Period* ^*^TRF interaction (Figure 2F). *Post-hoc* analyses revealed that during the Feeding period measurement dark fed animals had lower net insulin AUC as compared to light fed animals (*p* = 0.0089). Additionally, only light-TRF animals showed no significant diurnal difference in insulin AUC.

### Seventeen Hours Fasting in *ad libitum* Fed Animals (Experiment-2)

**Figure 3:**
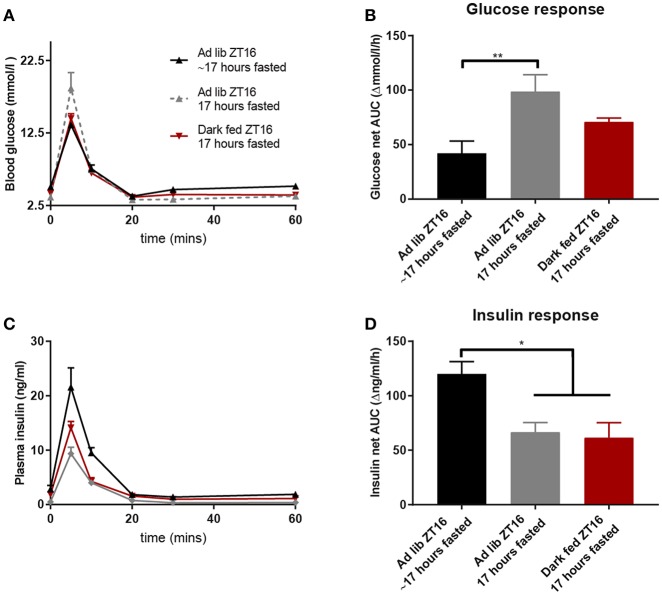
Glucose and insulin values during the ZT16 ivGTT of the 3 groups that were fasted for 17 h [either absolute or relative (~)]: the d-TRF group, the Ad lib group from Experiment-1 that was relatively fasted for ~17 h and the absolute 17 h fasted Ad lib animals from Experiment-2. **(A,C)** Glucose and insulin values during the ivGTT. **(B,D)** Net AUC (i.e., negative AUC [“undershoot”] subtracted from the positive AUC) of glucose and insulin responses, respectively. *N* = 8–9 animals per experimental group. ^*^p < 0.05, ^**^p < 0.01.

To better understand the effects of fasting status we compared the data of the dark fed animals at ZT16 (absolute fast of 17 h) with those of the *ad libitum* animals at ZT16 of Experiment-1 (relatively fast of 17 h as they could eat until ZT11) and 17 h-fasted AL animals of Experiment-2 (absolute fast of 17 h). Figure 3 shows that an absolute 17 h fast in AL animals caused an impaired glucose tolerance at *t* = 5 min (Figure 3A) and an increased net AUC (Figure 3B). Finally, the 17 h absolute-fasted, AL and TRF animals had a smaller insulin response as compared to the relative fasted AL animals as well as a smaller net AUC (Figures 3C,D).

## Discussion

We here present evidence that the well-known diurnal variation in glucose tolerance is due to both an intrinsic daily variation as well the preceding feeding/fasting condition. As expected animals fed *ad libitum* showed higher glucose tolerance at the beginning of the activity period than at the beginning of their sleep period. In neither TRF group, such a significant diurnal variation was observed, clearly indicating that the well-known diurnal variation is not only due to a difference in the feeding/fasting condition. Moreover, light fed animals showed no diurnal variation in either their glucose or insulin response, another clear indication that the normal diurnal variation is not the single result of either the feeding/fasting status or the circadian timing system. Of all the ivGTTs performed, the smallest insulin response was observed in dark fed animals during their ZT16 test. This improved glucose tolerance of the dark fed animals at ZT16 was due to a combined effect of time-of-day and prolonged fasting, as it was also observed in the *ad libitum* animals of Experiment-2 when fasted for 17 h and tested at ZT16, but not in the *ad libitum* animals of Experiment-1 with a relative fast of 17 h and tested at ZT16. Improved glucose tolerance was also not observed in the day fed animals, neither when fasted for 17 h and tested at ZT4, nor when fasted for 5 h and tested at ZT16. Overall, these results show that the normal daily variation in glucose metabolism induced by the circadian timing system can be enhanced when a strict and prolonged fasting period is imposed, but only when the timing of feeding and fasting behavior is strictly in line with the circadian timing system. The latter meaning fasting during the regular sleep period and only feeding during the regular wake period, thus, respectively, light and dark period for nocturnal animals.

Surprisingly, the disadvantageous effects of chronically feeding at the wrong time-of-day for glucose metabolism were quite limited. The most disadvantageous effect of daytime TRF being a higher insulin peak during the ivGTT in the fasting period and a slightly higher glucose response during the feeding period. Most likely, in the current experimental set-up the negative effects of feeding at the wrong time-of-day were counteracted by the positive effects of a long fasting period and the chronic condition of our TRF protocol (4 weeks) that allowed sufficient time for adaptation to the new rhythm in systemic glucose availability, through mechanisms that still need to be elucidated. One such potential mechanism that could be explored in future studies is corticosterone signaling as it is well-known that corticosterone (or cortisol in humans) affects glucose metabolism. Besides that, plasma corticosterone levels show a clear day/night rhythm with peak levels at the beginning of the active phase in both humans and rats [reviewed in (12)] and corticosterone levels may show an additional surge in TRF protocols just before food becomes available (13, 14). However, so far we have no indications that the presently used experimental set-up induces profound changes in daily plasma corticosterone levels (15). Another possible mechanism behind these changes in glucose metabolism is a change in the expression and localization of the insulin-dependent glucose transporters GLUT1 and GLUT4. Both Glut1 and Glut2 mRNA levels are downregulated in the heart tissue of streptozocin-induced diabetic rats, whilst the rhythmic expression of Glut1 was shifted in the cerebellum, clearly demonstrating the link between glucose transporters, circadian rhythms and diabetes (16). Previous experiments from both our group as well as others have shown that in skeletal muscle Glut4 mRNA expression and protein levels can fluctuate throughout the day, although this seems to differ depending on the specific muscle and animal model studied (17–20). Moreover, TBC1D1, a protein that regulates translocation of GLUT4 to the cell membrane has consistently been shown to be expressed rhythmically on both the mRNA and protein level, as well as displaying rhythmic phosphorylation statuses (19, 20). As the muscle clock is altered by TRF and the muscle clock controls glucose uptake and metabolism via e.g., GLUT4 translocation this could provide a mechanism for the changes observed, as skeletal muscle is the most prominent glucose-consuming tissue type in mammals (21).

Four weeks TRF during the active phase improved the glucose tolerance at ZT16, probably due to the prolonged fasting period. Indeed, the two groups with a strict fasting period of 17 h showed a smaller insulin response than the AL animals with a 17 h relative fasting period (Figures 3C,D). However, even with the same strict fasting duration, the same ZT and a highly similar insulin profile, glucose was taken up from the general circulation strikingly faster in the dark fed animals compared to the acutely fasted *ad libitum* animals, as indicated by the significant lower glucose levels at *t* = 5 as well as an overall lower glucose profile. Although the AL animals from Experiment-1 with a 17 h period of relative fasting showed an even lower glucose response (Figures 3A,B), they needed much more insulin for this (Figures 3C,D), indicating lowered insulin sensitivity. Thus, our results imply that a longer fasting period increases insulin sensitivity and reduces glucose clearance rates, but in the chronic condition the impaired glucose clearance returns to normal values again, while the improved insulin sensitivity is maintained.

### TRF and Other Shift-Work Models in Rodent Studies

Oral glucose tolerance in the light phase after a 14 h fast was worsened when mice were subjected to a shift-work paradigm with either 1 or 3 rotating night shifts (i.e., an inverted L:D cycle) per week for a duration of 3 weeks (22). In rats several risk factors such as increased abdominal fat, increased fasting glucose and increased glycemia in the light phase during an OGTT were found after 60 days of inverted feeding (i.e., 20% of caloric intake during the active phase and 80% of caloric intake during the inactive phase) (23). In line with our results, TRF during the active phase in mice improved glucose homeostasis and insulin sensitivity for several different nutritional challenges, even when TRF was applied only during weekdays and not during the weekend (8). Another study in mice found that 2 weeks of TRF for 12 h in the inactive phase resulted in only mild changes in fasting blood glucose rhythms, especially when compared with TRF in diabetic *ob/ob* mice (24). Although the study protocols discussed here greatly differ from each other, they all seem to confirm that animal models of shift-work impair glucose metabolism. However, it should be stressed that in many mice studies using TRF or other models of shift-work, differences in food intake and/or body weight are found between the experimental groups. As body weight and adiposity are important factors influencing insulin sensitivity, interpreting the direct effects of TRF on glucose metabolism are difficult when body weights or food intake differ between groups. In our experimental set-up, no significant differences were found between the groups for either food intake or body weight, which could possibly explain the limited negative effects of eating at the wrong time-of-day in our light fed group. Although several mice studies and one rat study reported increased body weight and adiposity after feeding at the wrong time-of-day (13, 37, 38), several other studies in both rats and mice reported varying effects on body weight, including no significant effects on body weight as well as decreases [reviewed in (5)]. Nevertheless, overall our results are in line with other rodent studies as they confirm that shift-work/TRF negatively affects glucose metabolism, although especially in mice studies these effect may be enhanced by changes in body weight and adiposity. Furthermore, our results also confirm the clear need for reporting the time-of-day and fasting duration in (rodent) studies on glucose metabolism in general.

### On the Effects of Prolonged Fasting Duration

In Experiment-2 we found an impaired glucose tolerance together with a lowered insulin response after acute and absolute fasting for 17 h in *ad libitum* fed animals. Similarly, in mice an overnight fast of 18 h resulted in enhanced insulin sensitivity as compared to a 5 h fast as measured with a hyperinsulinemic-euglycemic clamp (25). Another study in mice found that compared to a 4 h fast a 16 h overnight fast resulted in increased muscle insulin sensitivity, without changes in hepatic insulin sensitivity in the inactive phase (26). In a different series of experiments from our group rats acutely fasted for 17 h showed an even greater impaired glucose tolerance and decreased insulin response when tested at ZT4 as compared to ZT16 (Supplemental Figure 1). This difference in glucose responses after a similar fasting length is explained, for the largest part, by the major difference in prior feeding. For a GTT performed during the active phase (i.e., ZT16) it means that most of the fasting period was during the inactive phase, i.e., the animals' regular sleeping and fasting phase and animals are only deprived of 20–30% of their daily intake. However, for a GTT performed at the beginning of the inactive phase (i.e., ZT4) this means that most of the fasting period occurs during the active phase, i.e., the animals' regular awake and feeding phase and animals are deprived of 70–80% of their daily intake. Additional difference of course is the time-of-day, with daily glucose tolerance being higher at ZT16 than at ZT4 (Figure 1). Concluding, prolonged fasting, such as during our several-week exposure to TRF, reduces glucose tolerance, especially when measured in the inactive period.

### Shift-Work and TRF in Human Studies

Several studies in humans have been conducted to investigate the effects of shift-work on glucose metabolism. One study in healthy rotational shift-workers (nurses) found that post-prandial glucose concentrations were higher during a simulated night-shift as compared to simulated day-shift, accompanied with a lower insulin response during the first hour of the meal test (27). Also, β-cell responsivity was lowered during the night shift when compared to the day shift. It is not clear yet whether this decreased β-cell responsivity was an effect of the shift work or simply reflects the normal diurnal pattern found in β-cell function (28). In agreement with the study of Sharma (27) is another shift-work study in which the behavioral cycle was inverted (circadian misalignment) without altering the L/D schedule, by scheduling a recurring 28 h “day.” In 10 healthy adults (50% female) it was found that short-term circadian misalignment lead to increased postprandial glucose and insulin levels after a mixed meal test (29). In a similar experiment by the same group 9 healthy chronic shift-workers (67% female) underwent a mixed meal test at 8 AM and 8 PM, both during the aligned and misaligned conditions in order to dissect the independent effects of behavior and circadian timing (30). Under non-misaligned conditions, the normal daily variation was found, but circadian misalignment increased postprandial glucose levels and decreased insulin sensitivity. In a new series of experiments by the same research group and with the same experimental design the circadian misalignment paradigm was combined with the oral minimal model method in order to circumvent the long fasting durations and glucose level manipulations that are required for clamping (31, 32). By combining circadian misalignment with the oral minimal model Qian et al. (31) found that the circadian timing system and circadian misalignment both affect glucose tolerance, but through different mechanisms. While the circadian phase seems to mainly affect β-cell responsivity (quantified through c-peptide and glucose levels), circadian misalignment (i.e., behavior) seems to mainly lower estimates of insulin sensitivity and/or glucose uptake. Another recent study used a similar short-term misalignment protocol with 14 healthy young lean men (33). In agreement with the previous circadian misalignment studies, Wefers et al. (33) found that short-term circadian misalignment resulted in decreased muscle insulin sensitivity, without alterations in hepatic insulin sensitivity.

Several studies also implemented a form of TRF in humans. In a recent study male pre-diabetic patients underwent a GTT after TRF (3 meals during a 6 h period) as well as after the control condition (3 meals during a 12 h period) (34). Both the control and TRF interventions lasted for 5 weeks and food intake by the participants was rigorously controlled according to the authors. When the subjects were tested in the morning after an overnight fast there were no differences in fasting glucose or glucose profile during a 3-h oral GTT. However, after TRF insulin release was decreased and β cell responsiveness was increased during the oral GTT. Thus, similar to our study, strictly enforced TRF during the active phase for several weeks mainly affected insulin release during a GTT performed in the active phase and after an overnight fast. Similar to our Experiment-2, another human study comparing the traditional overnight fast with a prolonged fast of 36 h found that during the prolonged fast glucose tolerance during an OGTT was impaired together with a reduced insulin response (35). Another human study found decreased basal plasma glucose and insulin levels during a hyperinsulinemic-euglycemic clamp after a 36 h fast, with glucose levels lowering even more when the fast was prolonged to 60 h (36). These results are in line with our findings showing lower baseline glucose and insulin levels after a 17 h fast compared to a 5 h fast. Additionally, in the experiment by Hoeks et al. (36) whole-body insulin sensitivity was reduced after the prolonged fasting period and this was mainly accounted for by reduced insulin stimulated glucose disposal, indicating that mainly muscle glucose uptake and not hepatic insulin sensitivity was affected.

Overall, the human studies mimicking shift-work seem to find more profound disturbances than the animal studies mimicking shift-work. Main difference is that most human studies used short-term interventions, usually lasting <2 weeks, whereas the animal experiments usually use protocols of several weeks. The prolonged protocols likely allow the animals' sufficient time to (partly) adapt to the initial disturbances caused by circadian misalignment or shift work. The results from Experiment-2 where we fasted *ad libitum* fed rats acutely for 17 h are also in line with this idea. The changes seen after an acute 17 h fast period clearly differ from those of the TRF animals that were fasted for 17 h. By experimental design the TRF animals were daily fasted for 14 h, whereas *ad libitum* fed animals usually only have a daily fasting period of 6–8 h at the beginning of the light phase. During the TRF protocol especially glucose tolerance seems to improve again, i.e., although the insulin response is still reduced (Figure 3D), glucose uptake increases (Figure 3B).

## Conclusion

As recently nicely demonstrated in humans (29, 30), the present results clearly show that also in rats the well-known daily variation in glucose tolerance and insulin sensitivity is not only due to the daily variation in feeding condition, but also has a circadian component. In case the daily variation in glucose tolerance would have been completely dependent on the prior feeding/fasting condition, it would be expected that the daily variation in glucose and insulin responses in dark fed animals would be comparable to those of *ad libitum* animals, whereas those of light fed animals would be the reverse (i.e., 12 h shifted). On the other hand, in case the daily variation would have been completely dependent on the endogenous timing system, a similar daily variation would have been observed in all three groups as they were all housed in the same L/D condition. In fact, we observed that the daily variation in glucose responses was lost in both the dark and light fed group, whereas the daily variation in insulin responses was enhanced in the dark fed animals, but lost in light fed animals. Thus, together our results show that both time-of-day and the feeding/fasting condition modulate the effective glucose tolerance. It should be noted, however, that during ivGTTs insulin sensitivity is measured indirectly. Therefore, we cannot rule out the possibility that the smaller insulin release we find in our groups that are absolutely fasted for 17 h are due to e.g., impaired β-cell functioning, although without a change in insulin sensitivity that would conflict with the similar glucose clearance we find in these groups.

Surprisingly, feeding restricted to the inactive phase only marginally affected glucose tolerance and the concomitant insulin response compared to *ad libitum* conditions. Probably this “lack-of-effect” is due to the chronic condition of our experiment allowing sufficient time to adapt to this new situation, as well as the positive effects of the structurally enforced prolonged fasting period. For future experiments, it will be necessary to investigate how rapid and persistent these effects are when animals are switched back and forth between TRF and *ad libitum* feeding conditions, as such a situation more closely resembles shift work in humans. On the other hand, feeding restricted to the active phase *did* improve insulin sensitivity, but only during the active phase. Most likely this effect is due to the long fasting period coinciding with the regular sleep and fasting phase. Thus, together our results show that TRF in line with the circadian timing system enhances/strengthens the normal day/night difference in glucose tolerance and improves glucose tolerance when most needed, i.e., during the regular wake and feeding period. This also means that when applying TRF for therapeutic means eating should be restricted to the prescribed eating period, as glucose tolerance is worsened outside the regular eating hours.

## Data Availability

The datasets generated for this study are available on request to the corresponding author.

## Author Contributions

PdG and AK orchestrated the research project, performed the analyses, and wrote the manuscript. PdG, EF, WR, and NK performed the surgeries and the experiments. AK and C-XY provided both intellectual and financial support and helped writing and revising the manuscript.

### Conflict of Interest Statement

The authors declare that the research was conducted in the absence of any commercial or financial relationships that could be construed as a potential conflict of interest.

## Supplementary Material

Supplemental Figure 1 Control experiment in which 2 groups of animals were tested both in the 5 h fasted and 17 h fasted condition (with a wash-out period of 1 week in between measurements) either at ZT4 or ZT16. Net AUC of glucose **(a)** and insulin **(b)** responses during the GTTs, respectively. For glucose significant differences were found in the net AUC between the fasting conditions and the ZTs, as well as a significant interaction between Fasting condition and ZT (*p* < 0.0001 for all measures; two-way ANOVA). For insulin significant effects were found in the net AUC for the fasting conditions as well as for the ZTs (*p* < 0.0001 for both measures; two-way ANOVA). *N* = 7–9 animals per experimental group per measurement. ^*^*p* < 0.05, ^***^*p* < 0.001, ^****^*p* < 0.0001.
