# Supplementary figures and images for: Time-Restricted Feeding Improves Glucose Tolerance in Rats, but Only When in Line With the Circadian Timing System
Source: Front Endocrinol (Lausanne). 2019 Aug 21;10:554. doi: 10.3389/fendo.2019.00554 (PMC6712481; doi:10.3389/fendo.2019.00554)

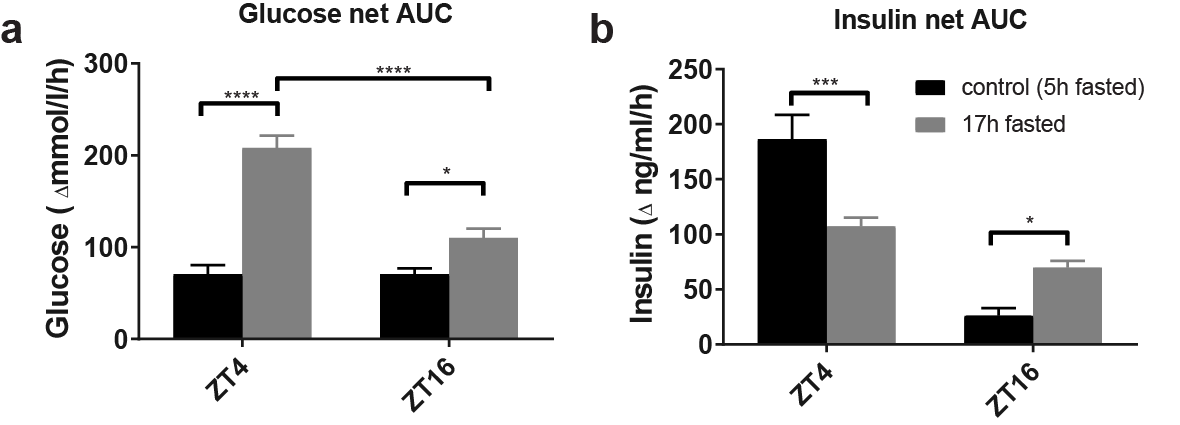

Supplement: Supplemental Figure 1 — Control experiment in which 2 groups of animals were tested both in the 5 h fasted and 17 h fasted condition (with a wash-out period of 1 week in between measurements) either at ZT4 or ZT16. Net AUC of glucose (a) and insulin (b) responses during the GTTs, respectively. For glucose significant differences were found in the net AUC between the fasting conditions and the ZTs, as well as a significant interaction between Fasting condition and ZT (p < 0.0001 for all measures; two-way ANOVA). For insulin significant effects were found in the net AUC for the fasting conditions as well as for the ZTs (p < 0.0001 for both measures; two-way ANOVA). N = 7–9 animals per experimental group per measurement. *p < 0.05, ***p < 0.001, ****p < 0.0001. [file Image_1.TIF]
